# Patellar Tendon Properties and Lower Limb Function in Rheumatoid Arthritis and Ankylosing Spondylitis versus Healthy Controls: A Cross-Sectional Study

**DOI:** 10.1155/2013/514743

**Published:** 2013-06-05

**Authors:** Verena Matschke, Jeremy G. Jones, Andrew B. Lemmey, Peter J. Maddison, Jeanette M. Thom

**Affiliations:** ^1^School of Sport, Health and Exercise Sciences, Bangor University, Bangor LL57 2DG, UK; ^2^Department of Rheumatology, Betsi Cadwaladr University Health Board, Llandudno Hospital, Llandudno LL30 1LB, UK; ^3^CERN, European Organization for Nuclear Research, 1211 Geneva 23, Switzerland

## Abstract

*Objective*. Rheumatoid arthritis (RA) and ankylosing spondylitis (AS) lead to inflammation in tendons and peritendinous tissues, but effects on biomechanical tendon function are unknown. This study investigated patellar tendon (PT) properties in stable, established RA and AS patients. *Methods*. We compared 18 RA patients (13 women, 59.0 ± 2.8 years, mean ± SEM) with 18 age- and sex-matched healthy controls (58.2 ± 3.2 years), and 12 AS patients (4 women, 52.9 ± 3.4 years) with 12 matched controls (54.5 ± 4.7 years). Assessments with electromyography, isokinetic dynamometry, and ultrasound included quadriceps muscle force and cross-sectional area (CSA), PT stiffness, and PT CSA. Additionally, measures of physical function and disease activity were performed. *Results*. PT stiffness and physical function were lower in RA and AS patients compared to healthy controls, without a significant difference in force production. PT CSA was significantly larger leading to reduction in Young's modulus (YM) in AS, but not in RA. *Conclusion*. The adverse changes in PT properties in RA and AS may contribute to their impaired physical function. AS, but not RA, leads to PT thickening without increasing PT stiffness, suggesting that PT thickening in AS is a disorganised repair process. Longitudinal studies need to investigate the time course of these changes and their response to exercise training.

## 1. Introduction

Chronic autoimmune arthritides are characterised by joint inflammation and progressive joint destruction and are accompanied by impaired physical function [1]. Inflammation also affects other musculoskeletal structures including tendons and their insertions into bone (entheses), but whether this leads to chronic alterations in the biomechanical function of the tendon-muscle complex is unknown. 

The function of a tendon is determined by its stiffness, that is, its elastic properties, which in turn influence skeletal muscle force output and function. When the force of the contracting muscle is transmitted via the tendon, the resulting elongation of the tendon attenuates the impact of the contraction on the connected bone. The force output is thereby reduced by a small amount, but this is stored as elastic energy and released on relaxation of the muscle [2]. Thus, this mechanism plays an essential part in the efficient performance of complex movements. Tendon properties also influence joint stability and the ability to make postural adjustments [3] and consequently play a major role in maintaining balance and preventing falls. In exercise physiology, ultrasound is used to investigate the biomechanical properties of healthy tendons (especially the load-bearing patellar and achilles tendons) and how they adapt to high intensity exercise, immobilisation, and changes with ageing [3–5]. In the elderly and after immobilisation, alterations in collagen content and cross linking lead to reduced tendon stiffness and size with a consequent reduction in collagen fibril diameter and number [6, 7].

Ankylosing spondylitis (AS) and rheumatoid arthritis (RA) are autoimmune inflammatory arthropathies with distinct pathology. An inflammatory process involving the entheses, that is, the tendon insertions to bone, is characteristic for AS [8]. The enthesis is the site where stress is concentrated in the tendon-muscle complex and therefore is prone to microdamage [9]. It is assumed that genetic factors in spondylarthropathies such as HLA-B27 lead to preferential deposition of adjuvant molecules derived from bacteria at the damaged enthesis, followed by abnormal tissue repair responses [9]. These in turn lead to thickening of the tissue and fibrocartilage formation at the tendon insertions (enthesophytes) and ligaments (such as syndesmophytes in the axial skeleton) and account for the gradual ankylosing of joints and vertebrae with loss of movement. Corresponding structural alterations found on magnetic resonance and ultrasound (US) imaging include thickening and hypervascularity of the tendon and enthesis [10, 11]. While enthesitis is the primary feature of AS, a secondary inflammatory reaction in the synovium and tenosynovium can occur [9]. 

In contrast, in RA the joint synovium is the primary antigenic target. Local diffusion of inflammatory cells and molecules from the synovium is thought to be responsible for inflammatory changes seen in and around adjacent tendons RA [12, 13]. The close proximity of the patellar and achilles tendons to the synovial spaces of the knee and ankle joint facilitates their direct exposure to the local inflammatory process. Enthesitis has also been demonstrated in these tendons in RA in connection with the joint synovitis [14]. It is thought that the high mechanical load that the patellar and achilles tendons undergo predisposes to this process, since entheseal involvement is not usually seen in other tendons of RA patients [14]. 

The primary aim of this research was to investigate the biomechanical properties of the human patellar tendon (PT) in the context of chronic inflammatory arthritis *in vivo*. Secondarily, we aimed to determine whether RA and AS have different effects on tendon size and function and therefore conducted two separate studies comparing stable RA patients with matched healthy controls, and stable AS patients with matched healthy controls. To our knowledge, this is the first time *in vivo* assessment methods of biomechanical PT properties with ultrasound have been applied to populations with arthropathies. Additionally, assessment of muscle size, muscle specific force (muscle force normalised to muscle size), and neural activation of the muscle with electromyography was performed.

## 2. Methods

### 2.1. Participant Characteristics and Disease Activity

Eighteen patients with RA according to the American Rheumatism Association 1987 revised criteria [15] and 12 patients with AS according to the European Spondylarthropathy Study Group criteria [16] were recruited from the rheumatology outpatient clinics of the local health board, as were, respectively, 18 and 12 age- and sex-matched healthy volunteers. Inclusion criteria for all patients were: disease duration of at least three years and stable disease activity (i.e., no flare or change in medication for the past three months). Exclusion criteria were the presence of any other catabolic disease, high dose steroid therapy (i.e., >10 mg prednisolone daily) or a recent steroid injection, and joint replacement or current pain or swelling in the right knee. The study was approved by the local research ethics committee and conducted in compliance with the Helsinki declaration.

Disease activity was assessed in RA patients by the modified Rheumatoid Arthritis Disease Activity Index-(RADAI-5) [17] and in AS patients by the Bath Ankylosing Spondylitis Disease Activity Index (BASDAI) [18]. RADAI-5 measures global RA disease activity over the previous six months and current disease activity in terms of swollen and tender joints, arthritis pain, general health, and duration of morning stiffness. BASDAI measures AS disease activity of the past week in terms of fatigue, spinal pain, peripheral joint pain and swelling, areas of localised tenderness (e.g., at the site of tendons and ligaments), and duration and severity of morning stiffness. Both RADAI-5 and BASDAI are scored from 0 = no disease activity to 10.

### 2.2. Habitual Physical Activity and Physical Function

A questionnaire on habitual physical activity [19] was administered to all participants, with separate scores (1 = sedentary to 4 = vigorous physical activity) for work and leisure time summed to a final score of 2–8. Objective physical function of the lower body was assessed by the 30-second chair sit-to-stand, the 8-foot-up-and-go [20], the 50-foot-walk, and single-leg balance tests [21]. The Modified Health Assessment Questionnaire (MHAQ) [22] and the 36 questions of the Short-Form Health Survey (SF-36) [23] provided information on subjective physical function and health-related quality of life (QoL), respectively. These questionnaires and physical function tests have been used in RA and AS populations before [24–28].

### 2.3. Setup for Quadriceps Muscle and Patella Tendon Measurements

**Figure 1:**
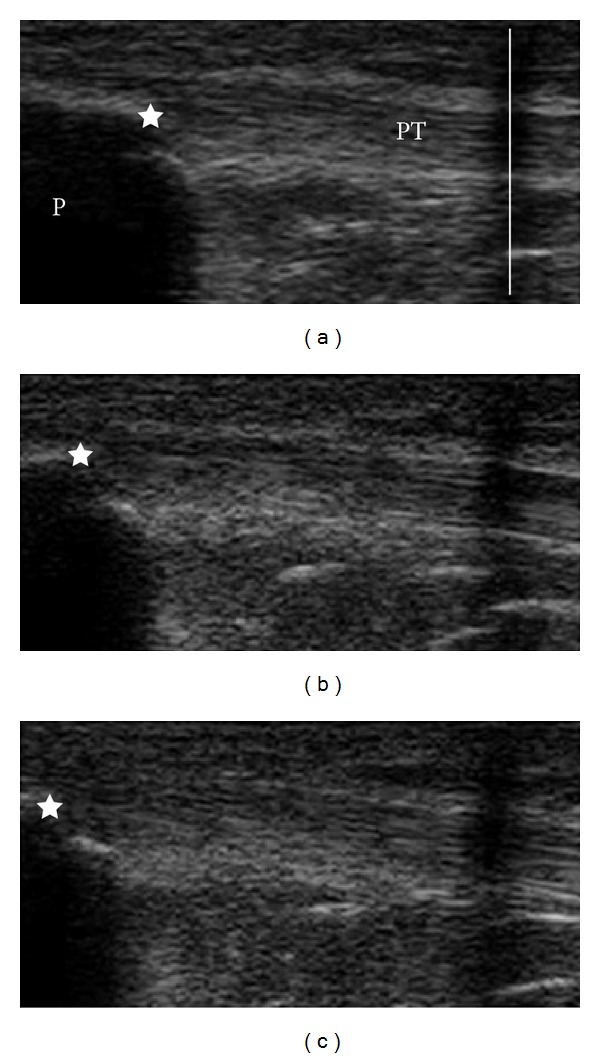
Illustration of patellar (P) tendon (PT) elongation. PT elongation shown from a skin marker (vertical line) to the tendon insertion at the patella (star) using US at rest (a) and during contractions ((b) and (c)).

Participants sat upright on an isokinetic dynamometer (CSMI Medical Solutions, Stoughton, MA, USA) with their right leg strapped to the dynamometer arm above the ankle. Additional straps were secured to prevent extraneous movement at the hips and shoulders. The knee joint angle was fixed at 90° from full leg extension and the hip angle at 90° [29]. PT stiffness was then determined using the method of Onambele-Pearson and Pearson [2]. After a set protocol of warm-up contractions, participants performed three ramped maximal voluntary isometric knee extension contractions (MVC), building up to maximum force with increasing effort over 4-5 seconds. During these contractions, participants crossed their arms over their chest to avoid the addition of arm muscle force to the quadriceps force measurements. Verbal encouragement was given. The US 7.5 MHz linear probe (MyLab50, Esaote, Firenze, Italy) was positioned sagittally over the PT and three video clips were recorded of PT excursion from the proximal and the distal attachments of the tendon to the bone, respectively (Figure 1). An external marker was fixed on the skin to detect accidental movement of the probe against the skin; when this occurred, recordings were repeated. The recordings were aligned by synchronization of force and US data. Real-time display of muscle torque on a computer screen provided feedback to the participants, and at least 1 minute rest between each MVC helped to minimise fatigue. US images were analysed using digitizing software (ImageJ, NIH, Bethesda, MD, USA), with the assessor blinded to the participant's disease status.

### 2.4. Calculation of Quadriceps Muscle Force

The MVC with the highest torque was used for analysis. Calculation of quadriceps muscle force accounted for torque, PT moment arm length [2], and antagonist cocontraction (which was estimated from electromyographic (EMG) activity [2, 30]). EMG activity (root mean square of the raw EMG signal) was recorded through self-adhesive Ag-AgCl electrodes (Ambu, Denmark) over the vastus lateralis (VL) and the long head of the biceps femoris (BF) during the extension MVCs and during three subsequent maximal isometric knee flexions. The latter data were used to correct extension torque for the effect of knee flexor muscle cocontraction.

The following equations were used to calculate quadriceps force: 

Quadriceps force = (BF torque + knee extension torque)/estimated PT moment arm length [30] where BF torque = (BF EMG during knee extension/BF EMG during knee flexion) × knee flexion torque [31]. 

### 2.5. Calculation of Patellar Tendon Stiffness

The tendon force-elongation relationship was assessed at intervals of 12.5% for maximal force development and fitted with second-order polynomial functions forced through zero [2]. Tendon stiffness for each participant was calculated at the level of maximum force of each individual's maximum force from the slope of the tangent (first derivative of the polynomial function) at this force level.

### 2.6. Patella Tendon Length, Patella Tendon Cross-Sectional Area, and Young's Modulus

PT length was defined as the distance between the apex of the patella and the superior aspect of the tibial tuberosity visualised on sagittal-plane ultrasound images with the knee joint at 90°. Three ultrasound images taken in the axial plane at 25%, 50%, and 75% of the patella tendon length were averaged to determine PT cross-sectional area (CSA). The mean CSA measurements at all levels were then averaged for the calculation of Young's modulus (YM = (tendon stiffness) × (tendon length/tendon CSA)) [5].

### 2.7. Quadriceps Muscle Cross-Sectional Area and Muscle Specific Force

To estimate muscle size, quadriceps anatomical CSA (ACSA) was measured by ultrasonography at 50% of the muscle length [30]. ACSA was measured separately for each of the four muscles of the quadriceps (VL, vastus medialis VM, vastus intermedius VI, rectus femoris RF) and then summed. In this way, noncontractile tissue between the muscles was not included in estimations of muscle tissue. Muscle specific force was calculated by normalising force to quadriceps ACSA. 

### 2.8. Statistics

All statistical analyses were performed using SPSS software v. 14.0 (Chicago, IL, USA). Depending on normality of distribution of the data, differences between the patient and matched control groups were determined by either Student's paired *t*-test or Wilcoxon test. Unless otherwise stated, values are presented as means ± SEM. Significance was accepted at the level *P* < 0.05.

## 3. Results

### 3.1. Participant Characteristics and Disease Activity

The anthropometric characteristics of the participants are summarised in Table 1. All patients had stable disease with low disease activity scores (DAS). 

In the RA group, 8 patients were rheumatoid factor positive, disease duration was 14 ± 2.3 years, and DAS by RADAI-5 was 3.3 ± 0.4. With regard to treatment with disease-modifying antirheumatic drugs (DMARDs), 15 RA patients (83%) were taking methotrexate (MTX), 2 in combination with an antitumour necrosis factor (TNF) agent (1 infliximab, 1 adalimumab), and 1 in combination with sulfasalazine (SSZ), 1 patient was on SSZ monotherapy. Eight patients (44%) were also taking a nonsteroidal anti-inflammatory drug (NSAID) and 5 (28%) prednisolone (range 1–10 mg/day; average dose 7 mg). 

In the AS group, disease duration was 20.7 ± 3.9 years and DAS by BASDAI 3.0 ± 0.6. Three patients were on DMARDs (1 MTX, 2 SSZ), two in combination with an NSAID, one was on the anti-TNF agent etanercept, five were on an NSAID only, and three required no medication for their arthritis. Five patients had conditions that are typically associated with spondylarthropathy: ulcerative colitis (*n* = 1), Crohn's disease (*n* = 1), and psoriasis (*n* = 3). AS patients were significantly shorter than their healthy counterparts, which is a consequence of the axial involvement of the disease leading to kyphosis and loss of body height, this explains the apparent large BMI of the AS patients. 

### 3.2. Habitual Physical Activity and Physical Function

There were no significant differences in habitual physical activity levels between either the patient groups or their respective matched controls (Table 2). Despite this, objective physical function was significantly reduced in RA patients (8-foot up-and-go by 17.2%, 50-foot walk by 25.7%, and one-leg standing balance by 27.4%) and in AS patients (sit-to-stand by 25.4%, 8-foot up-and-go by 15.8%, 50-foot walk by 19.5%) compared to their controls (Table 2). Similarly, both patient groups scored lower on subjective, self-assessed physical function, measured by mHAQ and by the SF-36 physical component summary (PCS) score. In addition, the AS group scored lower on psychological QoL factors from the SF-36 mental component summary (MCS) score than its matched control group (Table 2).

### 3.3. Patella Tendon Properties

**Figure 2:**
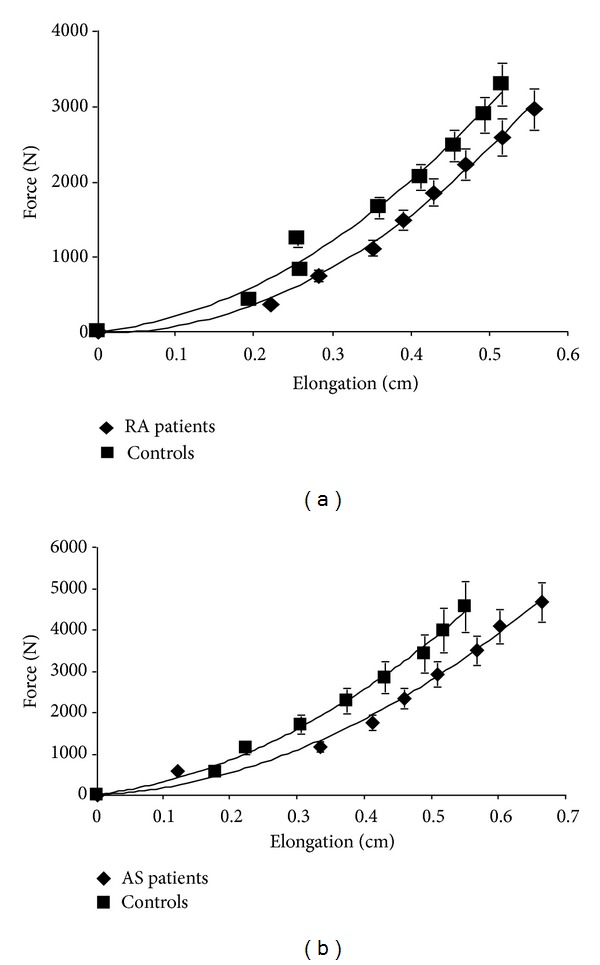
PT force-elongation relationship in RA patients (*n* = 18) (a) and AS patients (*n* = 12) (b) and their respective matched controls. Results are presented as means ± SEM.

Figure 2 shows increased elongation of the PT of the patient groups relative to their respective control groups at defined force levels, as demonstrated by a right shift of the force-elongation curves of the patient groups, indicating a reduction in tendon stiffness (i.e., the gradient to the curve). The calculated PT stiffness was significantly reduced in both the RA and AS patients compared to their controls (Table 3). This is consistent with the interpretation of the force-elongation curves. However, while the PT CSA of the RA group and their healthy control group was similar, it was increased in AS patients compared to their controls. There were no differences in PT length between the patient groups and their controls. Young's Modulus, which normalises PT stiffness to PT CSA, was therefore reduced in AS patients, but not in RA patients, relative to healthy controls.

### 3.4. Quadriceps Muscle Cross-Sectional Area and Muscle Specific Force

There were no differences in quadriceps muscle force or CSA between RA and AS patients and their respective matched controls. Consequently, muscle specific force was not compromised for either patient groups (Table 3).

## 4. Discussion

This study is to our knowledge the first to investigate the physiological properties of patellar tendons in patients with stable RA or AS. Compared to healthy age- and sex-matched controls, tendon stiffness in both patient groups is significantly reduced, and whereas the size of the PT was unchanged in RA, there was PT thickening in the AS group, resulting in pronounced reduction of YM. Despite preserved muscle force and size, these changes in tendon properties were accompanied by significant impairments in physical function. 

The reduction in PT stiffness is likely due to local and systemic effects of cytokines on the tendon, since proinflammatory cytokines are known to alter tendon structural characteristics in inflammatory arthropathies. The main drivers of the local inflammatory process are TNF-*α*, interleukin-1 (IL-1) and IL-6 which produce proteolytic enzymes such as matrix metalloproteinases that lead to collagen destruction [32], and the proangiogenic vascular endothelial growth factor, which evokes synovial hyperplasia and infiltration of macrophages and T cells into synovium [14]. According to the different pathologies of RA and AS inflammatory molecules target primarily the enthesis in AS, whereas in RA tendon involvement is thought to be secondary through the proximity to inflamed synovium [10, 11]. Systemically circulating cytokines [33] could have an additional detrimental effect on the tendon in both RA and AS. 

In addition to the effects of inflammation, disuse can be a contributor to reduced PT stiffness due to chronic reduction of the loading of the tendons [4, 34]. In the current study, however, there were no differences in habitual physical activity levels between the patient groups and their controls. It is therefore unlikely that disuse was causing the differences we observed in PT stiffness.

Tendon mechanical properties are essential for proprioception and for the reflex responses involved in rapid adjustment of muscle tension to positional changes [3], as well as the storing of elastic strain energy which is key to efficient locomotion. The reduced PT stiffness observed for both our patient groups was accompanied by significantly impaired physical function, despite no differences in muscle strength or size. This is in agreement with previous data that found that the decline in postural stability in the elderly correlated with reduced gastrocnemius tendon stiffness and YM [3]. The underlying biomechanical explanation is that increased compliance of the tendon reduces muscle fascicle length changes in response to passive joint movements and thereby impairs recognition of small movements by the muscle spindle [35].

 The finding that the PT CSA was increased in the AS patients but not the RA patients reflects the difference in the pathologies. The enthesis where a disorganised repair process takes place is the primary target organ in AS [9]. With MRI imaging, McGonagle et al. demonstrated characteristic entheseal inflammatory changes of perientheseal swelling and oedema and bone marrow oedema associated with knee synovitis in spondylarthropathies. These changes are not seen in RA, in particular, adjacent to entheseal insertions [36]. Histological findings from cadavers indicate the underlying structural changes at and around the enthesis including periosteal bone reaction, alterations of the bone structure, and increased bone formation, endochondral ossification, and vascular invasion of the fibrocartilage that facilitates access for inflammatory cells [37]. Our results are in agreement with Balint et al. who demonstrated thickening on US of the infrapatellar and tibial entheseal insertions in patients with SpA [38], and with other authors describing tendon thickening on US in SpA [11, 39].

A further indication of the ineffective repair process in the tendon in AS is the fact that although PT CSA was increased in our AS patients this did not attenuate loss of PT stiffness, and resulted in the strikingly low YM. In RA, YM was not reduced significantly because of the unchanged tendon size. This emphasizes the differences in structural adaptive responses of the tendon in these different conditions.

In ageing, a different process leads to degenerative changes of the tendon, with variable effects on tendon size having been demonstrated in tendon CSA with age [5, 40, 41]. Increases in tendon stiffness in healthy individuals following exercise training have been primarily attributed to intrinsic adaptations of the tendon material properties [5, 42]. These adaptations and additional increases in PT CSA with exercise [5, 40] (e.g., a heterogeneous, region-specific increase of PT CSA at the enthesis) possibly provide protection to the stressed tendon [42, 43].

As the current study was cross-sectional in design, our results do not provide information on the time course of tendon changes. However, in a case report on unilateral inflammatory knee effusion in a patient with newly diagnosed RA [28], we found reduction of PT stiffness initially only in the leg affected by knee joint effusion, but one year later both PTs were affected despite controlled disease activity and maintenance of regular physical activity. Loss of muscle specific force and muscle CSA in the affected leg in the acute stage of knee effusion was also observed. Whilst muscle specific force and muscle size showed signs of partial recovery following resolution of the joint effusion by intraarticular corticosteroid injection and stabilisation of disease activity, there was no recovery of the PT biomechanics. This corresponds to the results now presented in moderately physically active patients with controlled, established RA and AS, where tendon stiffness is reduced. Previous publications showed that whereas stable RA patients are characterised by attenuated muscle mass and consequently reduced physical function [44, 45], their muscle specific force and activation capacity are preserved [26, 27]. 

In RA, high intensity exercise has been shown to restore muscle quantity, strength and function [25, 33, 44–46]. Similarly, high intensity exercise training may be required to achieve beneficial adaptations of tendon properties. In healthy populations, this form of exercise is associated with increases in tendon stiffness and rate of force development [5, 42]. Additionally, intensive exercise training has been shown to reverse the loss of tendon stiffness consequent of either immobilization or ageing [5, 30, 34, 47]. In particular, eccentric exercise, which is characterised by high frequency fluctuations of force and transfers higher loads through the tendons than concentric exercise, has shown clinical effectiveness in tendinopathies [48] and is thought to promote tendon remodelling through increased cross-linking of collagen fibres [49]. Intermittent loading has been shown to reduce inflammation in tendon tissue *in vitro* [50], and thus it is possible that eccentric exercise would be beneficial for tendons affected by inflammatory arthropathies. Future studies should investigate the response of RA and AS to tendon-specific training.

There are several limitations to our study. Firstly, higher participant numbers would have been helpful to clarify if, in the context of the loss of tendon stiffness, the YM in the RA group would have reached significantly low levels. Secondly, although we assessed disease activity through patient questionnaires and inflammatory markers in the blood, we did not have an objective measure of the local inflammation of the PT or enthesis. Both MRI and US can provide a detailed assessment of tendinopathic features in different regions along the tendon and at the enthesis; however, we had no access to a clinician trained in clinical ultrasound or MRI evaluation of tendinous structures. Similarly, histological data on the inflammatory processes in the tendon alongside our tests would have enhanced understanding of the relationship between inflammation of the tendinous and peritendinous structures and their biomechanical properties. This was judged to be unjustifiably invasive. A possible future project could assess tendon biomechanical properties in patients with RA and AS awaiting tendon surgery, whereby biopsy material could be gained without inconveniencing patients. Finally, a more detailed assessment of proprioception in future studies could further elucidate the functional implications of tendon abnormalities in RA and AS.

## 5. Conclusions

In summary, the present study reveals that PT properties are adversely affected in RA and AS and possibly contribute to the disability associated with these conditions. The demonstration of different changes in tendon structure add to our increasing understanding of the differences between the pathologies of RA and AS. Tendinopathies can be asymptomatic and therefore may go unnoticed in the context of inflammatory arthropathies. However, further research is needed to elucidate the role of tendon properties in the impact of chronic arthropathies, and to develop and evaluate treatments for preserving and restoring function of the muscle-tendon complex. 

## Figures and Tables

**Table 1:** Participant anthropometric characteristics. Presented are the data (mean ± SEM) of RA patients (*n* = 18; 13 women) and their age- and sex-matched healthy controls (*n* = 18) as well as the data of AS patients (*n* = 12; 4 women) and their age- and sex-matched controls (*n* = 12).

	RA study			AS study		
	RA patients (*n* = 18)	Healthy controls (*n* = 18)	*P* value	AS patients (*n* = 12)	Healthy controls (*n* = 12)	*P* value
Age (years)	59 ± 2.8	58 ± 3.2	0.35	53.7 ± 3.3	54.8 ± 3.3	0.68
Height (m)	1.65 ± 0.01	1.69 ± 0.03	0.21	1.66 ± 0.03	1.74 ± 0.02	<0.001
Weight (kg)	75.1 ± 3.3	73.8 ± 3.2	0.98	79.0 ± 4.2	78.2 ± 2.9	0.89
BMI	27.4 ± 1.0	26.0 ± 1.2	0.61	28.7 ± 1.07	25.7 ± 0.9	0.08

**Table 2:** Habitual physical activity and subjective and objective physical function. Presented are the results (mean ± SEM) of RA (*n* = 18; 13 women) and AS (*n* = 12; 4 women) patients and their respective age- and sex-matched healthy controls.

	RA study			AS study		
	RA patients	Healthy controls	*P* value	AS patients	Healthy controls	*P* value
	(*n* = 18)	(*n* = 18)		(*n* = 12)	(*n* = 12)	
Habitual physical activity (range 2–8)	4.9 ± 0.7	4.4 ± 1.4	0.17	4.6 ± 0.4	4.3 ± 0.3	0.46
30-sec and sit-to-stand (*n*)	12.8 ± 0.8	13.7 ± 0.4	0.3	11.8 ± 0.7	15.8 ± 1.1	**0.002**
8-foot-up-and-go (sec)	6.0 ± 0.3	5.2 ± 0.2	**0.03**	5.3 ± 0.3	4.6 ± 0.2	**0.03**
50-foot-walk (sec)	9.2 ± 0.5	7.4 ± 0.3	**0.004**	7.9 ± 0.4	6.6 ± 0.4	**0.03**
One-leg balance (cumulative) (sec)	50.2 ± 5.6	66.9 ± 6.3	**0.04**	74.9 ± 4.5	78.7 ± 6.2	0.62
mHAQ (range 0–3)	0.60 ± 0.06	0.18 ± 0.03	**<0.001**	0.56 ± 0.12	0.17 ± 0.0	**<0.01**
SF-36 physical component (range 22–59)	38.1 ± 3.3	51.3 ± 1.9	**<0.001**	41.6 ± 3.6	50.7 ± 1.6	**0.04**
SF-36 mental component (range 11–62)	39.6 ± 2.8	43.2 ± 1.5	0.11	34.7 ± 3.1	44.3 ± 1.3	**0.03**

**Table 3:** Physiological data of RA (*n* = 18; 13 women) and AS (*n* = 12; 4 women) patients versus age- and sex-matched healthy controls. ACSA: anatomical cross-sectional area. Results are presented as mean ± SEM.

	RA study			AS study		
	RA patients	Healthy controls	*P* value	AS patients	Healthy controls	*P* value
	(*n* = 18)	(*n* = 18)		(*n* = 12)	(*n* = 12)	
Quadriceps force (N)	3407 ± 301	3640 ± 244	0.44	4887 ± 492	4922 ± 494	0.93
Quadriceps ACSA (cm^2^)	62.7 ± 3.6	60.3 ± 2.7	0.40	77.4 ± 5.3	74.6 ± 4.9	0.62
Muscle quality (N/cm^2^)	55.1 ± 4.0	60.9 ± 3.4	0.30	61.6 ± 3.3	65.4 ± 4.2	0.34
Patella tendon stiffness (N/mm)	1017 ± 122	1385 ± 158	**0.04**	1131 ± 133	1751 ± 212	**0.01**
Patella tendon CSA (mm^2^)	91.4 ± 4.5	91.3 ± 2.6	0.89	111.8 ± 5.8	96.9 ± 3.9	**0.04**
Young's modulus (GPa)	0.59 ± 0.07	0.74 ± 0.08	0.13	0.49 ± 0.04	0.90 ± 0.10	**<0.001**
